# Upright multidetector CT with 320-row gantry: a technical innovation providing insights into human anatomy under gravity and potential clinical implications

**DOI:** 10.1093/bjr/tqaf196

**Published:** 2025-08-26

**Authors:** Masahiro Jinzaki, Minoru Yamada, Yoichi Yokoyama, Takehiro Nakahara, Takeo Nagura, Yoko Inamoto, Fumiko Yagi, Orito Ikeda, Mohammed Alshahri, Katsuhiro Mizutani, Yoshitake Yamada

**Affiliations:** Department of Radiology, Keio University School of Medicine, Tokyo 160-8582, Japan; Department of Radiology, Keio University School of Medicine, Tokyo 160-8582, Japan; Department of Radiology, Keio University School of Medicine, Tokyo 160-8582, Japan; Department of Radiology, Keio University School of Medicine, Tokyo 160-8582, Japan; Department of Orthopedic Surgery, Keio University School of Medicine, Tokyo 160-8582, Japan; Faculty of Rehabilitation, School of Health Sciences, Fujita Health University, Toyoake, Aichi 470-1192, Japan; Department of Radiology, Keio University School of Medicine, Tokyo 160-8582, Japan; Department of Radiology, Keio University School of Medicine, Tokyo 160-8582, Japan; Department of Radiology, Keio University School of Medicine, Tokyo 160-8582, Japan; Department of Neurosurgery, Keio University School of Medicine, Tokyo 160-8582, Japan; Department of Radiology, Keio University School of Medicine, Tokyo 160-8582, Japan

**Keywords:** multidetector CT, standing, posture, upright CT, vein, pelvic floor, voiding, workflow

## Abstract

CT performed in the supine position has been highly effective in diagnosing organic diseases such as cancer, arteriosclerosis, and infections, significantly contributing to increased life expectancy. In an ageing society, extending healthy life expectancy becomes more critical, requiring early diagnosis of functional disorders. We have led the industry-academia collaboration in developing an upright MDCT system. Although this system maintains the same physical specifications as conventional MDCT, it differs significantly in imaging configuration—allowing supine, upright, and sitting positions—and offers improved workflow while requiring only two-thirds of the installation space. Unlike conventional MDCT, it allows for the assessment of anatomical changes under gravity. It also enables the objective diagnosis and grading of functional diseases, in which findings were not apparent on conventional CT, and enable the study of the pathogenesis of functional diseases which worsen symptoms in the upright position. Furthermore, it allows for noninvasive evaluation of dynamic functions such as swallowing and voiding, which can only be assessed in standing or sitting positions.

## Introduction

CT was developed in the late 1960s and early 1970s^1^ and has been performed in the supine position for over 50 years. Its primary diagnostic targets have been organic diseases such as cancer, arteriosclerosis, and infections. Around 2000, the introduction of multidetector-row CT (MDCT) enabled faster imaging, higher resolution, and wider coverage,^2^^,^^3^ allowing for the detection of earlier-stage lesions and incidental findings, as well as more accurate staging of diseases. As a result, by facilitating precise diagnosis, CT has significantly contributed to extending life expectancy.

In recent years, as societies face super-ageing populations, extending not only life expectancy but also healthy life expectancy has become increasingly important. To achieve this, it is essential to evaluate and diagnose functional disorders at an early stage. Recognizing this need, we anticipated that a CT system capable of imaging patients in a standing position would be essential.

As a method for capturing 3-dimensional images in a standing position, cone-beam CT (CBCT) has been available. However, compared to MDCT, CBCT has several limitations: its gantry rotation speed per cycle is significantly slower (20-30 seconds), its spatial resolution is lower (slice thickness of 2 mm), and its gantry size is smaller, resulting in a limited imaging range.^4^^,^^5^ Additionally, CBCT has poor contrast resolution for soft tissues, restricting its use primarily to high-contrast, small-area imaging of structures like teeth and bones.

In contrast, MDCT can achieve a slice thickness of 0.5 mm, a gantry rotation speed of 0.25-0.275 seconds, a gantry size of 70-80 cm, and excellent visualization of soft tissues. With the advent of 64-detector-row CT scanners, it has become possible to image the entire body trunk in less than 20 seconds. This duration aligns with the time a person can maintain a stationary standing posture, making the concept of upright MDCT scanning a more realistic possibility.

**Figure 1.**
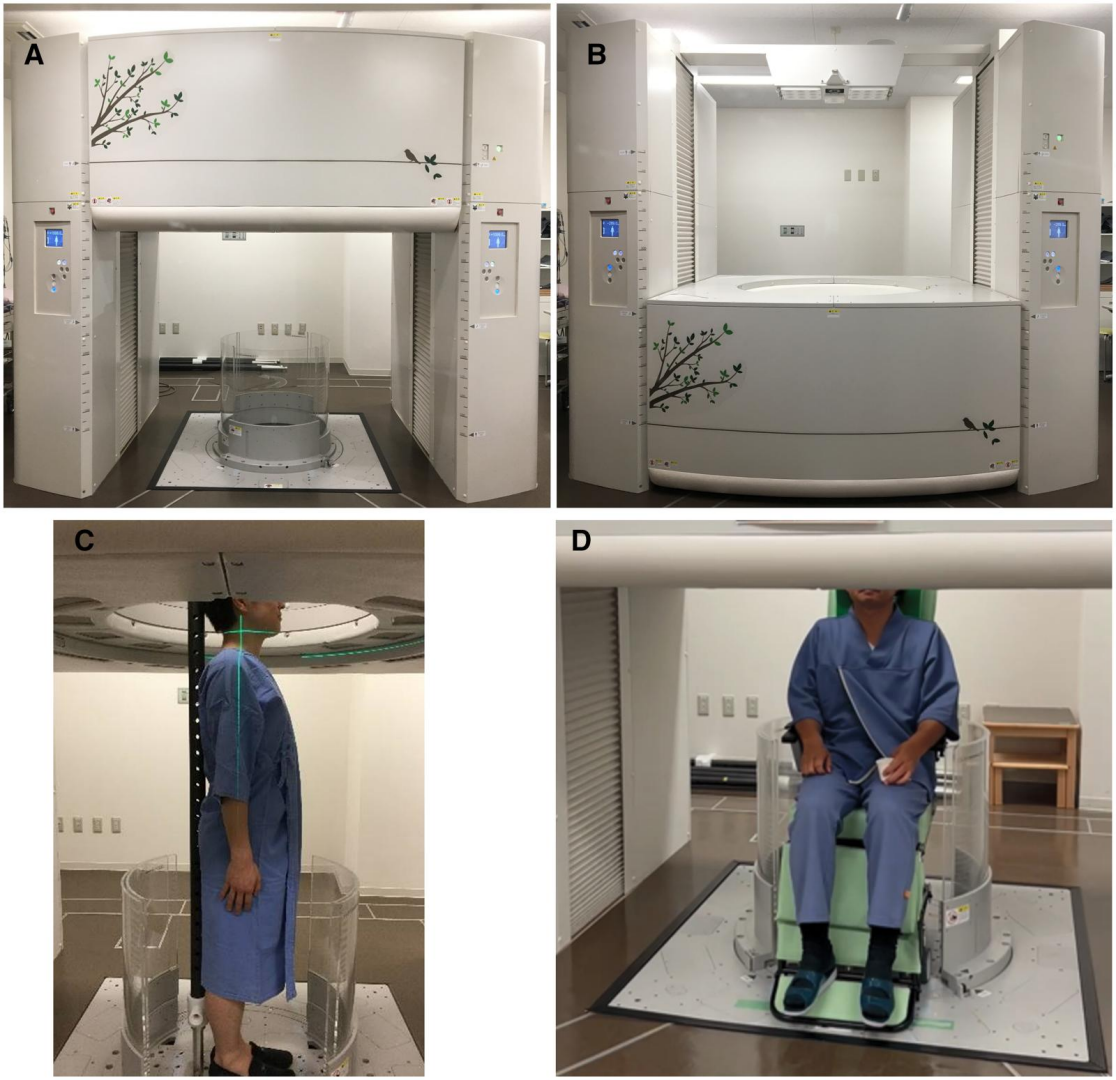
Upright MDCT machine and tools for safety. Our upright MDCT system enables up-and-down movements with a transverse 320 detector-row gantry. (A) Gantry in the up position. (B) Gantry in the down position. (C) Standing position with the use of pole to lightly support the back of the patient and knee-high acrylic wall encircling the body. (D) Sitting position.

We persuaded industry partners of the importance of this concept and have led the development of upright MDCT through an industry-academia collaboration.^6^ The first unit capable of full-body imaging in upright position and sitting position (Figure 1) was installed in our institution in 2017.

## Constitution of upright MDCT

Our upright MDCT system is capable of up-and-down movements of a transverse 320 detector-row gantry (0.5 mm in detector size), with a gantry rotation speed of 0.275 seconds and an optimal performance of 1200 views per rotation^6^ (Figure 1). This upright CT system allows imaging in either the 80 detector-row mode or the 320 detector-row mode. The gantry is supported by a stand on either side, ensuring stability during operation.

Table 1 summarizes the key technical specifications and configurations of the conventional 320-detector-row CT and the upright 320-detector-row CT. The 2 systems share essentially the same specifications. However, the configuration is quite different in the imaging position (supine vs upright and sitting) and the installation footprint, which is reduced to two-thirds in the upright CT system. Furthermore, in conventional MDCT, the gantry remains stationary along the body axis while the patient moves together with the table. In contrast, in upright MDCT, the gantry itself moves vertically along the body axis while the patient remains still. In upright MDCT, any vibration or deviation from a precise horizontal alignment during the vertical motion of the heavy rotating gantry can lead to image quality degradation. To address this challenge, the system incorporates advanced technologies such as a high-speed motion control system, a horizontal compensation mechanism, and a highly rigid rotating structure to minimize motion artifacts associated with gantry movement.

**Table 1.** Key technical specifications and configuration of conventional 320 detector-row CT and upright 320 detector-row CT.

	Conventional 320 detector-row CT	Upright 320 detector-row CT
Imaging position	Supine	Upright, Sitting
Detector configuration	0.5 mm × 320 or 80	0.5 mm × 320 or 80
Maximum gantry rotation speed	0.275 seconds	0.275 seconds
Beam pitch	0.673, 0.813, 1.388 @ 80 detector mode	0.673, 0.813, 1.388 @ 80 detector mode
kVp	80, 100, 120, 135 kVp	80, 100, 120, 135 kVp
mA	10∼600 mA	10∼600 mA
Bore size	780 mm	780 mm
FOV (field of view)	500 mm	500 mm
Reconstruction	AIDR 3D, SEMAR	AIDR 3D, SEMAR
Maximum bed speed	160 mm/s	N.A.
Maximum gantry movement speed	N.A.	100 mm/s
Technique to prevent motion artifact due to gantry movement	N.A.	High-speed motion control system Horizontal compensation mechanism Highly rigid rotating structure
Installation space		Two-thirds compared to conventional CT

Abbreviations: AIDR 3D = adaptive iterative dose reduction; SEMAR = single energy metal artifact reduction.

Also, for patient safety during scanning, the system is equipped with a pinch-prevention mechanism and a contact interlock control system. To stabilize patients in the standing position, a support pole was installed to provide gentle back support (Figure 1). Additionally, a knee-high acrylic barrier surrounds the patient to prevent falls (Figure 1).

## Performance of upright MDCT

### Physical characteristics

We evaluated the physical performance using phantoms to assess potential image quality degradation resulting from the up-down movement system in the upright MDCT. We compared spatial resolution, noise characteristics, and CT numbers between the upright MDCT and conventional 320 detector-row CT scanner. We found both the NPS curve and MTF curve of upright MDCT were comparable to that of conventional MDCT which obtains images in supine position (conventional MDCT).^6^ Additionally, the CT numbers of the upright MDCT were almost same with that of the conventional MDCT.^6^

### Improvement of workflow

With upright MDCT, patients do not need to lie on a bed and can be scanned standing for a more efficient examination. We measured the examination time which includes the patient entering/exiting time and the scanning operation time using healthy volunteers. The total access time (entry time and exiting time) was a half that of conventional MDCT.^6^ There was no significant difference in the scanning operation time between the 2 positions. The shorter time required for exams will allow more exams with 1 machine, which may contribute to efficiency in CT imaging.

### Safety and comfortability

We performed a questionnaire survey among healthy volunteers who underwent upright MDCT, asking them to rate safety and comfortability on a 5-point scale (0 = very dissatisfied, 5 = very satisfied). The results showed that the average scores for both safety and comfort exceeded 4, indicating that the examination method was well accepted and satisfactory for the subjects.

### Remote operation

One of the advantages of upright MDCT is that patients can enter the room and stand at the centre of gantry by themselves, without any aid by radiographer. Also, this machine can be remotely operated.

Thus, upright MDCT helps prevent radiographers from unintentionally contracting infections through direct contact with infected patients, even during a pandemic like COVID-19. Remote operation may be one of the important factors for the next-generation CT.

### Scanning protocol

For most cases, the helical scan was performed with the following parameters: gantry rotation speed of 0.275 seconds, tube voltage of 100 or 120 kVp, noise index (S.D.) of 18∼24, 0.5 mm × 80 detector mode, beam pitch of 0.813, field of view (FOV) of 500 mm, reconstruction kernel of F03, and reconstruction algorithm of adaptive iterative dose reduction (AIDR) 3D Enhanced.

For 4-dimensional dynamic imaging of the same region, scanning was performed in continuous or intermittent acquisition, with a gantry rotation time of 0.275 seconds, tube voltage of 120 kVp, tube current of 40 mA, 0.5 mm × 320 detector mode (a 16-cm coverage), FOV of 240 mm for swallowing, FOV of 500 mm for voiding, and reconstruction kernel of F03, and reconstruction algorithm of ADIR 3D Enhanced. The total radiation dose is 1.7mSv for swallowing and 3∼4mSv for urination.

## The effect of gravity on the human body

### Head region

Traditionally, the brain is considered a homeostatic organ that remains relatively stable in position under normal gravitational conditions on Earth (1G), with negligible effects of gravity on intracranial structures. However, reports on astronauts returning from microgravity environments have indicated upward brain displacement and ventricular enlargement,^7^^,^^8^ raising the question of whether intracranial structures are truly immobile under Earth’s gravity.

One study demonstrated that the position of intracranial structures does, in fact, shift with changes in body posture.^9^ Specifically, a 1.0 mm descent of the inferior cerebellar tonsil tip was observed in the sitting position compared to the supine position. Additionally, the pineal body exhibited a displacement of 0.7 mm in the caudal direction and 0.7 mm in the ventral direction in the sitting position. These findings provide critical insights into the extent of intracranial structural variation due to postural changes in healthy individuals, which is essential for establishing criteria to define abnormal brain positioning.

### Lung region

**Figure 2.**
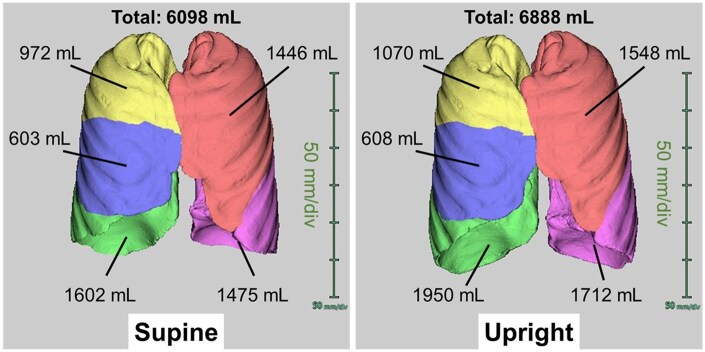
Volume-rendering conventional and upright MDCT images illustrating lung and lobe volume measurements during inspiration. The total lung volume increases in the upright position (right) compared to the supine position (left), but the volume of the middle lobe remains consistent (yellow: right upper lobe, blue: right middle lobe, green: right lower lobe, pink: left upper lobe, purple: left lower lobe).

In a cohort of healthy volunteers, lung volumes of both the upper and lower lobes, as well as airway volumes and luminal areas, were reported to be significantly greater in the standing and sitting positions compared to the supine position during both inspiration and expiration.^10^^,^^11^ Specifically, total lung volume increased by 9% and 10% in the inspiratory standing and sitting positions, respectively, and by 6.4% and 9.5% in the expiratory standing and sitting positions (Figure 2). However, the inspiratory right middle lobe volume remained consistent across all 3 positions, whereas the expiratory right middle lobe volume was significantly lower in the standing and sitting positions, showing a 16.3% and 14.1% reduction, respectively, compared to the supine position.^10^

Lung attenuation gradients in anteroposterior direction, which was observed in the supine position,^12^ disappeared in the standing position.^13^ However, the craniocaudal lung attenuation gradient, which was not present in the supine position, appeared in the standing position.^13^

Furthermore, studies involving both healthy individuals and patients with chronic obstructive pulmonary disease or idiopathic pulmonary fibrosis have demonstrated that lung volume and airway measurements obtained from upright MDCT exhibit stronger correlations with pulmonary function indices, including total lung capacity, vital capacity, functional residual capacity, residual volume, and forced expiratory volume in 1 second (FEV_1_).^10–11^^,^^14–16^ These findings suggest that upright MDCT may serve as a valuable tool for predicting pulmonary function test outcomes in clinical practice.

### Venous system

It is well known that venous diameter varies with body position. This can be easily verified by observing the jugular or lower limb veins using ultrasound while altering the patient’s posture, allowing for a quantitative assessment of the rate of change in venous diameter.^17–19^ However, there has been no method to accurately evaluate intrathoracic vessel size in the upright position.

**Figure 3.**
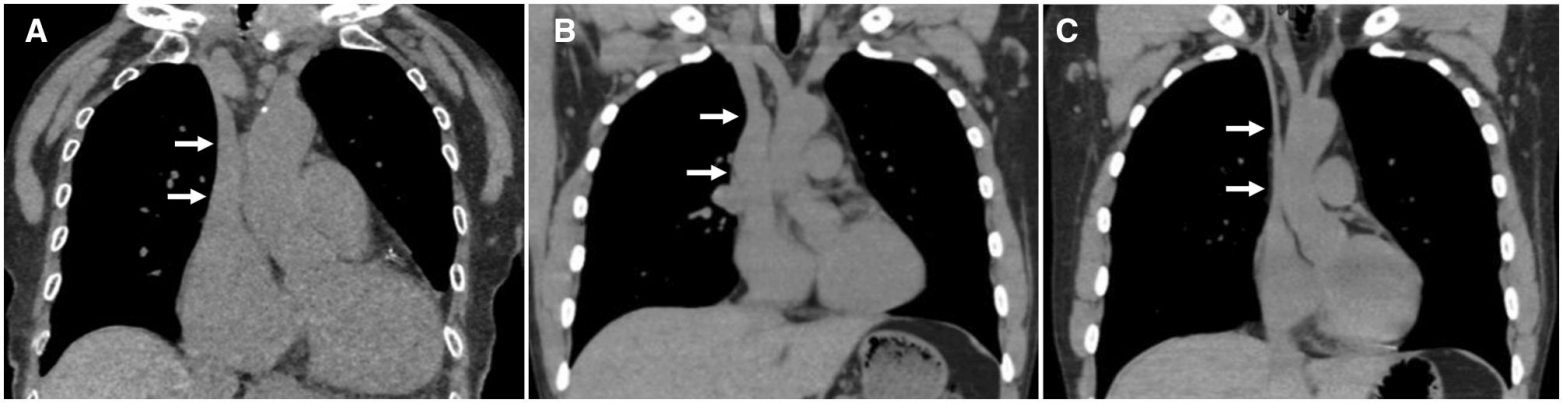
Change of the size of the superior vena cava (SVC) between the supine position and standing position and prediction of severity of heart failure. In healthy volunteers, compared to conventional MDCT in supine position (A), SVC diameter (→) is reduced by about 80% on upright MDCT (B) In patients with right heart failure (C), the superior vena cava does not shrink much in the standing position.

In a cohort of healthy volunteers, upright imaging revealed location-specific changes in the cross-sectional area of the vena cava. Compared to the supine position, the superior vena cava (SVC) area was significantly reduced by 80% in the standing position (Figure 3), while the diameter at the diaphragm level remained unchanged. In contrast, the inferior vena cava (IVC) area increased by 37% in the standing position.^6^ Notably, intracranial venous diameters were unaffected by postural changes.^20^ Interestingly, upon transitioning from supine to upright, the internal jugular vein above the heart collapsed, whereas posterior venous structures, such as the anterior condylar confluence and its tributaries, expanded. This redistribution led to a shift in dominant cerebral venous outflow from the internal jugular veins to the vertebral venous system.

Upright MDCT has further enabled non-invasive assessment of venous anatomy and physiology. It allows for the visualization of saphenous vein valves. Furthermore, the number of tributaries and the vessel area per leg were greater in the upright position than in the supine position,^21^ indicating its potential utility in preoperative graft evaluation for bypass surgery.

Recent studies have also highlighted the value of upright MDCT in haemodynamic assessment. For instance, when used to evaluate elevated right atrial pressure (>5 mmHg), upright MDCT measurement of the SVC yielded an area under the curve (AUC) of 0.91, outperforming the AUC of 0.78 obtained with supine MDCT^22^ (Figure 3).

### Pelvic floor

Upright MDCT is also valuable for assessing the degree of pelvic floor muscle relaxation in a standing position. Before the development of upright MDCT, few studies had evaluated pelvic relaxation in the standing position. One MRI-based study compared the pelvic floor position between supine and sitting postures but not standing and reported no significant differences in healthy individuals.^23^

In contrast, 1 study using upright MDCT in healthy individuals revealed that both the bladder neck (BN) and the anorectal junction (ARJ) decreased significantly in the standing position compared to the supine position (mean descent; 9.4 ± 6.0 mm for BN and 8.0 ± 5.6 mm for ARJ). Additionally, the extent of bladder displacement between the supine and standing positions was significantly greater in women than in men (12.2 ± 5.2 mm vs 6.7 ± 5.6 mm).^6^

The anatomical position of the pelvic floor also exhibits age-related changes that differ between males and females. In men, the distance between the BN and the pubococcygeal line (BN-PCL) increases with age in both the standing and supine positions. This upward shift may reflect age-related changes in enlarged prostate volume due to benign prostatic hyperplasia.^24^ Interestingly, the position of the ARJ in men does not show a statistically significant correlation with age in either posture.^24^ In women, the BN-PCL distance remains relatively stable with age in both standing and supine position. However, ARJ showed a significant tendency to descend with age particularly in the standing position. The female pelvic floor is designed to be more flexible than male pelvis floor for adaptation to pregnancy and childbirth.^24^

## Clinical application

### Objective diagnosis and grading of the functional diseases, in which findings were not apparent on conventional MDCT

The objective diagnosis and grading of these diseases, as well as assessments of the clinical significance of abnormal findings (in relation to patients’ symptoms), are essential for appropriate clinical care and accurate outcome studies. However, these diseases are not well evaluated in the imaging of supine position such as conventional MDCT, and thus, its evaluations largely depend on inspection or integrated interviews by experts.

#### Spondylolisthesis

**Figure 4.**
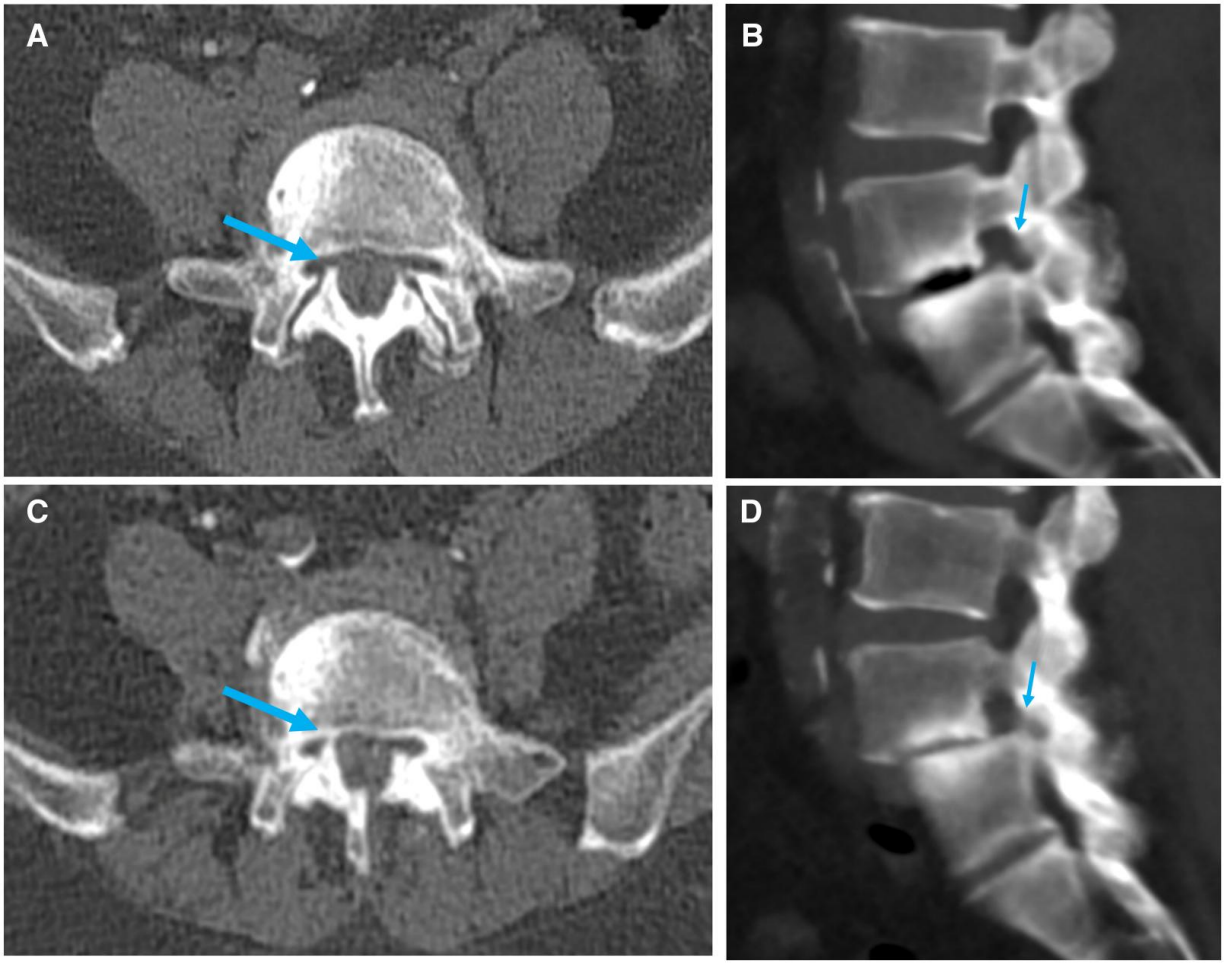
A case of spondylolisthesis. This patient had spondylolisthesis and suffered from low back pain only in the standing position. While no abnormalities were detected on axial (A) and oblique sagittal images (B) of conventional MDCT, upright MDCT revealed narrowing of the right neural foramen on axial (C) and oblique sagittal images (D). Following surgery, the patient’s back pain was relieved.

Spondylolisthesis is a spinal pathology frequently diagnosed in the elderly.^25^ This disease is often not well evaluated in the imaging of supine position.^25^ It is reported that supine MRI evaluation such as canal stenosis and disk herniation have high positive and high negative rates.^26–28^ Upright MDCT is useful for the evaluation of the patient, who suffered from back pain only in the standing position. Lumbar foraminal stenosis of spondylolisthesis often becomes more remarkable in the standing position^6^ (Figure 4). This change in the spinal canal space and foramen with posture explains the cause of intermittent claudication with lumbar spondylolisthesis.

#### Pelvic prolapse

Pelvic floor prolapse is a common genitourinary disorder affecting close to a third of all women over the age of 40.^29^^,^^30^ Since the decision of pelvic organ prolapse (POP) of whether to proceed with a transvaginal or a transabdominal approach is dependent on which of the organ is affected and the degree of prolapse as well as patient and surgeon preference,^31^ dynamic supine MRI evaluation was expected to provide integral information in the preoperative assessment. However, there are variations in the sensitivity of dynamic MRI for POP^32^^,^^33^ and dynamic MRI does not correlate well with clinical findings in patients with middle compartment prolapse.^34^

**Figure 5.**
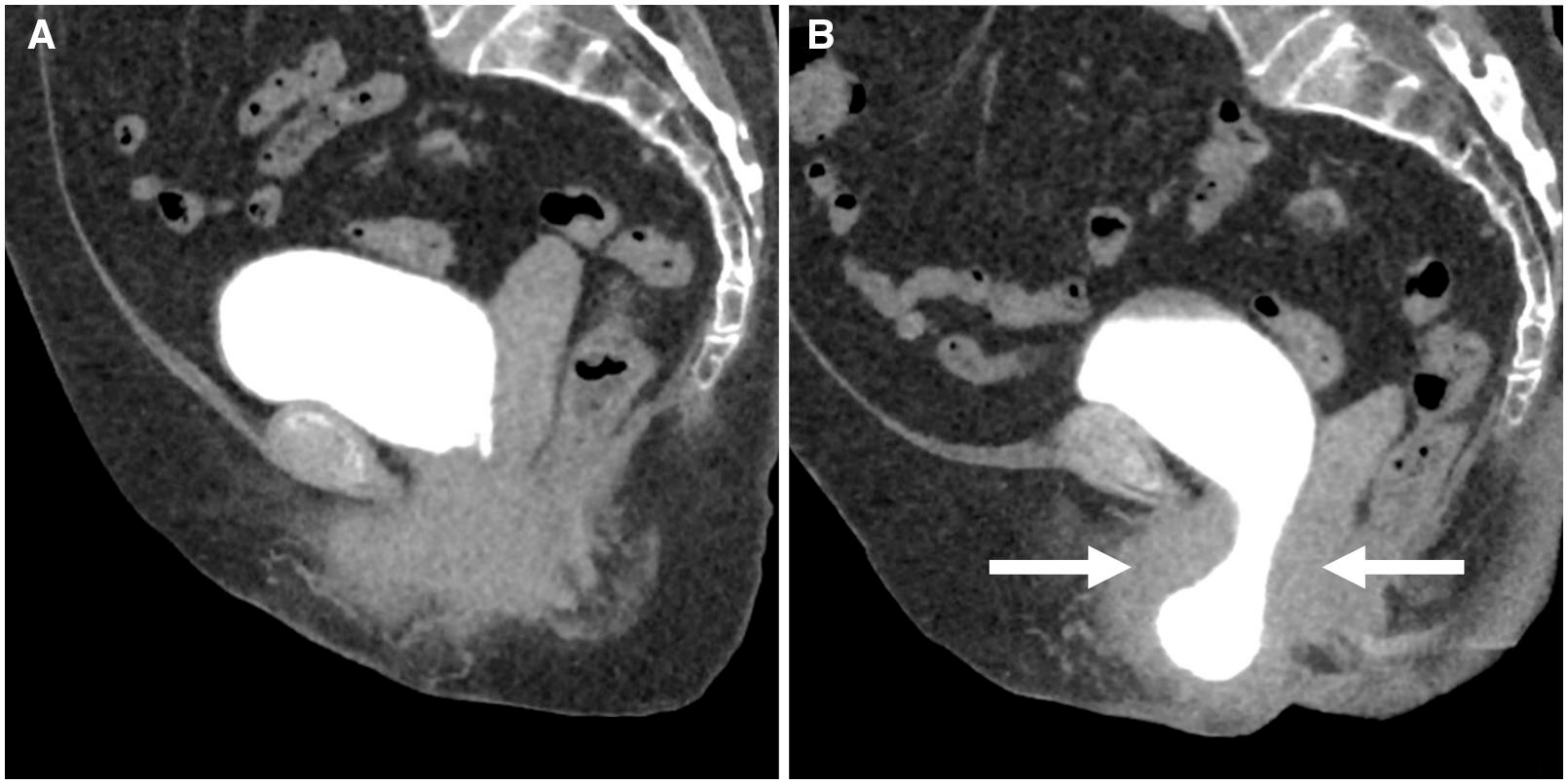
A case of pelvic floor prolapse. The bladder prolapse was not detected on conventional MDCT (A), but detected on upright MDCT (B: arrow).

The pelvic floor prolapses, which were not detected on conventional supine MDCT, are often detected on upright MDCT (Figure 5). Upright MDCT could enable the objective diagnosis and determine the clinical significance of pelvic floor prolapse, which has largely depended on expert experience.

Previous survey-based studies have reported that 40%-60% of older women experience symptoms suggestive of POP, indicating that the true prevalence of POP may be substantially higher than commonly recognized.^35–38^ However, due to embarrassment or the perception that these symptoms are simply part of ageing, many do not seek medical attention.^37^^,^^38^ Upright CT may provide a less intrusive and more acceptable diagnostic approach for POP, thereby facilitating timely recognition and appropriate clinical management.

#### Inguinal hernia

Inguinal hernia is a common condition among men that increases substantially with age.^39^ Randomized controlled trials of the inguinal hernia reported that watchful waiting for minimally symptomatic or asymptomatic type is safe.^40–42^ However, since the inguinal hernia often reduces when the patient lies down, conventional imaging has not been used for the preoperative assessment of inguinal hernia.^43^^,^^44^ Thus, the evaluations of these diseases largely depend on inspection or integrated interviews by experts in addition to conventional imaging.

**Figure 6.**
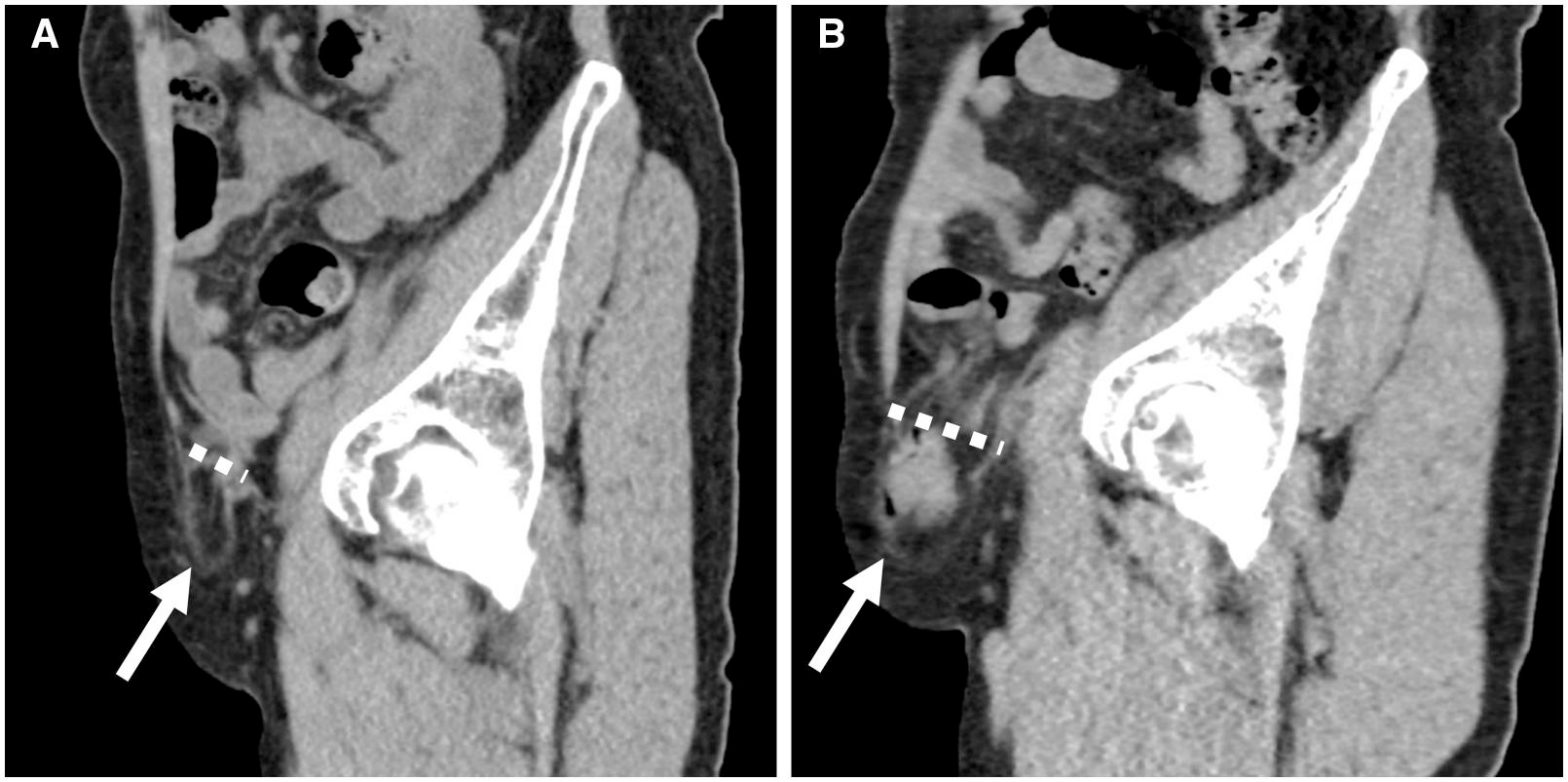
A case of inguinal hernia. The conventional MDCT (A) shows mild left inguinal hernia (dot line, size of hernia orifice is 16.0 mm) and the hernia content is fat tissue only (arrow). The upright MDCT (B) shows more apparent inguinal hernia (dot line, size of hernia orifice is 32.1 mm) and the hernia contents include small intestine and fat tissue (arrow).

Upright MDCT can more clearly depict inguinal hernias which are latent in conventional MDCT, with differences in the contents of the hernia sac and the size of the hernia orifice between the two. Upright MDCT allows for a more accurate diagnosis of hernia severity in a more natural posture (Figure 6).

#### Osteoarthritis

The classification of knee osteoarthritis (OA) is crucial for early diagnosis, as timely intervention can help prevent disease progression and irreversible joint damage.^45^ However, conventional 2-dimensional (2D) radiographs have limited sensitivity in detecting early-stage deformities, particularly in the tibiofemoral joint. The widely used Kellgren-Lawrence (K-L) classification, first introduced in 1957, is based on features such as osteophyte formation and joint space narrowing.^46^ Yet, subtle changes in these features are difficult to detect on 2D images, making early diagnosis challenging. Several studies have pointed out the limitations of the K-L system, especially for identifying early knee OA.^47–50^

To overcome these challenges, Oka et al^51^ developed a computer-aided diagnosis system known as knee OA computer-aided diagnosis (KOACAD), which automatically evaluates knee OA and provides both normal and threshold values for various radiographic parameters. However, KOACAD still has limitations. For example, the difference in joint space width between K-L grades 1 and 2 is less than 0.4 mm—too small to reliably distinguish using standard radiography.^52^ As a result, diagnosing early knee OA based solely on 2D imaging remains problematic.

Recent studies have explored the use of upright MDCT to evaluate 3-dimensional (3D) alignment changes in the knee during weight-bearing.^53^ In patients with end-stage knee OA, upright MDCT revealed significant 3D deformities (specifically, increased flexion, adduction, and tibial internal rotation) when comparing the standing position to the supine position. Interestingly, while no significant differences in flexion or adduction were observed between K-L grades 1 and 2, greater tibial internal rotation was seen in K-L grade 2 knees compared to grade 1 during standing. These results suggest that greater tibial rotation when weight-bearing is a key consideration to differentiate early-stage patients from advanced stage patients.^53^

### Clarifying the pathogenesis of functional diseases which worsen symptoms in the upright position

Upright MDCT enables visualization of gravity-induced anatomical shifts that may contribute to disease pathophysiology, offering novel insights into various conditions. Incorporating upright imaging into routine practice may enhance our understanding of position-dependent pathophysiology and improve surgical planning, postoperative management, and symptom correlation.

#### Head region

**Figure 7.**
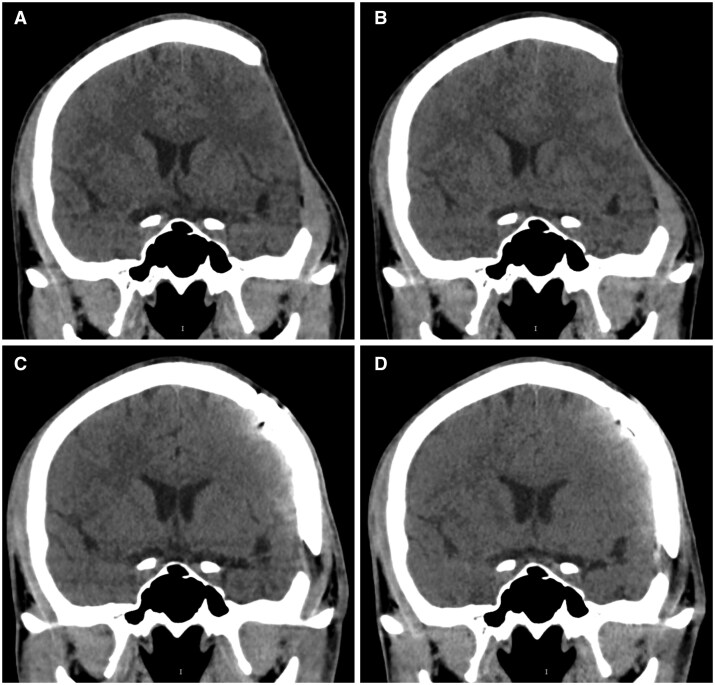
A case of sinking skin flap syndrome. A man with a left epidural haematoma and temporal bone fracture underwent frontotemporal decompressive craniectomy. Although neurologically intact while supine, he developed slight right hemiparesis, headache, and lightheadedness in the upright position. Compared to the supine scan (A), upright MDCT revealed significant brain surface and midline shift (B), consistent with his postural symptoms. The patient underwent cranioplasty with artificial bone, which resulted in complete symptom relief. Postoperative conventional (C) and upright MDCT (D) showed resolution of the positional brain shift.

Recent studies utilizing upright CT have provided novel insights into posture-dependent intracranial dynamics, particularly in post-surgical and cerebrospinal fluid (CSF)-related pathologies. In a cohort of 67 post-neurosurgical patients, upright CT identified clinically significant positional brain shifts (PBS ≥ 5 mm) in nearly one-third of cases. Supratentorial craniectomy, residual intracranial air, parenchymal brain injury, and large skull defects were major contributors.^54^ These findings suggest that upright CT is critical for risk assessment and safe mobilization in patients vulnerable to complications such as sinking skin flap syndrome (Figure 7). A related case report further illustrated this syndrome in a patient with postural hemiparesis. Upright CT revealed a marked brain shift not seen in the supine position, which normalized post-cranioplasty alongside clinical improvement.^55^ This highlights upright MDCT’s unique capacity to detect paradoxical herniation and midline shift, which can remain occult on conventional imaging (Figure 7).

In another context, upright MDCT demonstrated its diagnostic value in evaluating a supracerebellar arachnoid cyst associated with orthostatic headache.^56^ The scan revealed cyst enlargement and cerebellar descent in the upright posture, correlating with symptom provocation and emphasizing posture-dependent anatomical changes even in otherwise asymptomatic lesions.

Finally, in patients undergoing endoscopic endonasal skull-base surgery (EESBS), upright MDCT enabled quantitative assessment of skull-base displacement under postural changes.^57^ Intracranial shift of the reconstructed skull base, especially in cases with large dural defects or sphenoid sinus involvement, was found to potentially contribute to CSF leakage, a common postoperative complication.

One study reported that the degree of enophthalmos in patients with orbital floor fractures differs between upright and supine MDCT, highlighting the potential importance of evaluating globe position using upright MDCT, which reflects a more physiologic, daily-life posture, in the preoperative assessment.^58^

#### Thorax region

Recent evidence highlights the role of upright MDCT in elucidating posture-dependent cardiopulmonary impairment in thoracic deformities. In pectus excavatum, upright MDCT demonstrated a significantly reduced anterior-posterior thoracic diameter and increased Haller index compared to supine MDCT, likely due to anterior displacement of the lower thoracic spine.^59^ These morphological changes may underlie the posture-induced exacerbation of cardiopulmonary symptoms, as evidenced by a case showing pulmonary vein compression visible only in the upright position.

Similarly, in a patient with severe thoracic lordoscoliosis, upright CT revealed bronchial compression and a reduction in lung volume compared to supine imaging. Following posterior spinal fusion, upright MDCT showed both anatomical decompression and improvement in lung volume, which marks the perioperative visualization of upright lung function in spinal deformity correction.

### Dynamic evaluation of human function

With the use of a 320 detector-row mode, it is possible to acquire continuous imaging of the same region over time within a 16 cm coverage area. This scan mode enables the generation of time-resolved 3-dimensional imaging (that is 4-dimensional imaging), facilitating dynamic evaluations of physiological functions such as swallowing and urination.

#### Swallowing function assessment

A standardized method for analysing swallowing dynamics, known as swallowing CT, has been performed in a reclined sitting position. In this technique, patients consume food mixed with 5% w/v barium sulfate during the examination.^60–62^ This method offers the first 4-dimensional (4D) visualization of the swallowing process, enabling the simultaneous observation and analysis of swallowing-related kinematics that are difficult to assess with conventional imaging methods. Swallowing CT allows for quantitative analysis, including timing of swallowing events and kinematic measurements such as pharyngeal volume, the maximum opening area of the upper esophageal sphincter, and the trajectories of the hyoid bone and larynx.^63–67^ These capabilities make swallowing CT a valuable tool in clinical practice, research, and education. Moreover, it can be performed with a low radiation dose of approximately 2 mSv.

This detailed visualization of anatomical structures and their functions—often not clearly captured by traditional methods—has advanced our understanding of the complex physiology of swallowing, including mechanisms like laryngeal closure for airway protection. Several studies have provided insight into the timing and anticipatory adjustments of the true vocal cords during swallowing.^68–72^

Swallowing CT can also be used to validate findings from conventional 2-dimensional fluoroscopic evaluations.^73^^,^^74^ Clinically, by comparing 4D swallowing dynamics between healthy individuals and patients with dysphagia, it is possible to investigate the pathological mechanisms behind issues such as aspiration and pharyngeal residue. Additionally, this method helps identify which structures are more susceptible to functional decline, which functions are likely to improve with training, and which show high adaptability (plasticity) in response to rehabilitation.

**Figure 8.**
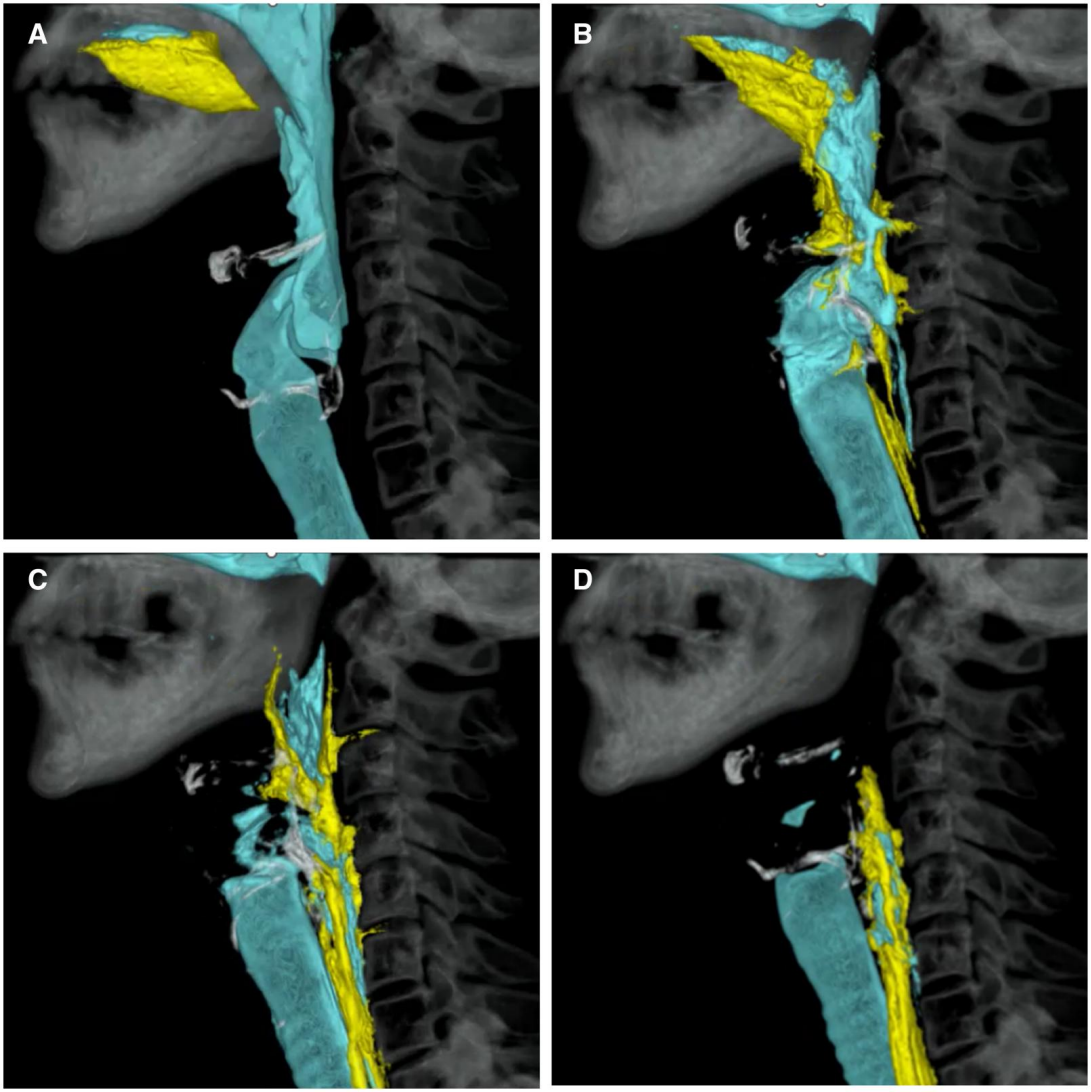
Temporal evaluation of swallowing function in 3 dimensions. By having the subject swallow barium-containing food (yellow) while seated inside the gantry of a upright MDCT scanner and continuously imaging the process (A-D), it is possible to evaluate swallowing function in 3 dimensions. The area in blue indicates the airways.

The recent introduction of upright MDCT has overcome previous limitations of performing the examination only in a reclined sitting position. This advancement brings swallowing CT closer to real-world application in both research and clinical settings (Figure 8). Based on these findings, we anticipate the development of personalized rehabilitation strategies targeting specific functional deficits, the ability to quantify the effectiveness of these interventions, and ultimately, the creation of preventive training programs for dysphagia.

#### Voiding function assessment

**Figure 9.**
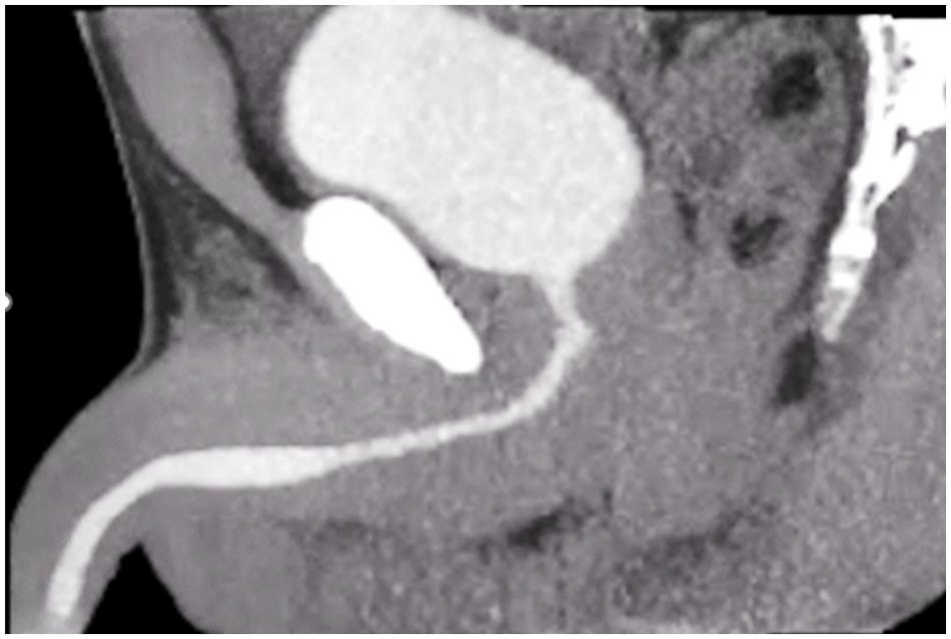
Temporal evaluation of voiding function in 3 dimensions. Contrast medium is injected intravenously and allowed to accumulate in the bladder. After 40 minutes, the patient is asked to void, and the voiding process is continuously imaged using upright MDCT. This enables noninvasive evaluation of voiding function.

Conventional methods for evaluating voiding function typically require retrograde injection of contrast medium into the bladder through the urethra, making the procedure highly invasive. To address this, we have developed a novel, non-invasive imaging technique that enables visualization of the voiding process using upright MDCT, with a low radiation dose of just 2-4 mSv (Figure 9). This method involves intravenous administration of contrast medium, followed by CT imaging approximately 40 minutes later, which is performed while the patient is actively voiding.

Using this approach, we have analysed both healthy individuals and patients with voiding dysfunction. In healthy individuals, the technique has helped clarify the normal mechanisms of voiding. In patients, it has revealed distinct pathological patterns, including (1) early closure of the entire urethra shortly after the start of urination, (2) localized closure at the urethral opening, and (3) reduced bladder contractility. These patterns correspond to specific treatment options, such as α-blockers, surgery, or anticholinergic medications.

Based on these findings, we believe that upright MDCT can serve as a valuable tool for guiding treatment strategies in patients with urinary disorders.

### Health screening

Upright MDCT offers significant clinical benefits, especially in the field of health screening. One of its main advantages is that patients can be scanned while standing, similar to a standard X-ray, without needing to lie down. This reduces physical burden on the examinee, increases patient throughput with a single unit, and greatly improves overall examination efficiency. This streamlined workflow is particularly well suited for health screenings, where the target population consists of healthy individuals who are generally more comfortable standing. In addition, for lung CT screenings, upright MDCT eliminates the increased density that often appears along the dorsal pleura in the supine position, making image interpretation easier for radiologists.^13^

In conclusion, we have developed upright MDCT, which enables imaging of subjects in the standing and sitting position. Upright MDCT offers the same physical capabilities as conventional MDCT, while improving workflow and ensuring patient safety and comfort. It also supports remote operation, a key feature for next-generation CT systems in terms of infection control.

Upright MDCT is particularly valuable for understanding the effects of gravity on the anatomy of the entire body. Clinically, it allows for the objective diagnosis and grading of functional diseases, which have traditionally relied heavily on expert interpretation. It may also help elucidate the pathophysiology of functional disorders whose symptoms worsen in the upright position. Furthermore, upright MDCT enables noninvasive evaluation of dynamic functions such as swallowing and voiding, which can only be accurately assessed in standing or sitting postures.

As we move into an era of super-ageing societies, the importance of promoting healthy longevity is becoming increasingly clear. In this context, early detection and quantitative assessment of functional decline, followed by appropriate intervention, are essential. While conventional MDCT has played a key role in extending life expectancy by focusing on the diagnosis of many organic diseases and injuries, upright MDCT holds the potential to contribute to extending *healthy* life expectancy.
